# Childhood Injury Prevention: Intervention in the Bedouin City of Rahat

**DOI:** 10.1100/tsw.2005.70

**Published:** 2005-08-08

**Authors:** Michal Hemmo-Lotem, Efrat Merrick, Liri Endy-Findling, Aziza Abu Freh, Claudia Jinich-Aronowitz, Liat Korn, Joav Merrick

**Affiliations:** ^1^ Beterem, National Center for Children's Health and Safety, Box 7050, IL-49170 Petach Tiqva, Israel; ^2^ School of Public Health, Haifa University, Haifa, Israel; ^3^ National Institute of Child Health and Human Development, Ben Gurion University of the Negev, Beer-Sheva, Israel; ^4^ Beterem, Rahat, Israel; ^5^ Center for Multidisciplinary Research in Aging, Faculty of Health Sciences, Ben Gurion University of the Negev, Beer-Sheva, Israel; ^6^ Office of the Medical Director, Division for Mental Retardation, Ministry of Social Affairs, Jerusalem, Israel

**Keywords:** childhood injury, emergency medicine, injury, epidemiology, public health, Israel

## Abstract

For several years, the National Center for Children's Health and Safety (Beterem) has worked on many levels to promote safety and prevent injury of the children in Israel. As part of intervention programs in 20 communities around Israel, this paper describes a 1-year, multidisciplinary, multistrategic childhood safety promotion and injury prevention project. The project took place in the Bedouin city of Rahat in the Southern part of Israel, the Negev, conducted by a local safety coordinator. This specific intervention study took place from March 2003 to March 2004. The main goal was to identify hazards and dangerous obstacles in public places in Rahat, then remove or repair the obstacles found, in order to secure a safe public environment for children. “Obstacle” was defined as any barrier that could endanger the safety of a child. Ten examples are used to illustrate this applied research project, and 80% of the problems were solved within the project period (time to solve between 1 week to 3 months, depending on various factors). We recommend the involvement of a safety coordinator from the community to focus on safety hazards for children, the use of a documentation diary to log the time frame, and also the use of pictures to illustrate the hazards and the changes, or to use as arguments in the lobbying process.

## INTRODUCTION

The National Center for Children's Health and Safety in Israel (Beterem) is committed to the vision of "making the world a safer place for children". Beterem's foremost mission is to enhance child safety and diminish the injury rates in Israel. Beterem is involved in many intervention projects around the country together with various partners and collaborators, with a special focus on villages and cities with limited resources. At the moment, more than 20 communities are supported by Beterem and this paper will present the assessment and intervention in the Bedouin city of Rahat in the south of Israel.

## BEDOUIN COMMUNITY IN ISRAEL

The Bedouins of the Negev are in transition from a nomadic life to more permanent settlements. They are an ethnic, religious, and cultural minority with many socioeconomic problems. Their language is Arabic and usually only the men are able to speak Hebrew. Many of the men are unemployed and their families live on social security income. The utilization of medical services offered by the health authorities is not fully used due to language, cultural, and economic difficulties, but also due to geographic scattering[1].

The term Bedouin is derived from the Arabic term *badiah* meaning “desert” and Bedouin Arab nomads have lived in the Negev since the 5$^{th}$ century AD at least. After a major exodus to other countries during the Israel War of Independence (1947–49), the number of Negev Bedouins has increased from 12,000 in 1948 to more than 100,000 today, comprising 24% of the Beer Sheva District by the end of 1995[2,3].

Israeli Bedouin society is in major social, economic, and cultural transition as a result of rapid semi-urbanization and sedentarization[4]. The sedentarization process, initiated by the Turkish Empire and subsequently by the British and the Israeli governments, has sought to relocate the Bedouins into planned settlements. The result has been stratified sedentarization forms, from semi-nomadic encampments mainly in the periphery, to spontaneous settlements and planned permanent urban towns and cities. The Bedouins in the Negev live in three types of settlements, as defined by the urbanization scale:

- *Planned permanent settlements*: Supported by the government, which receive municipal, health, and other modern services. Residents are generally characterized by a high level of sedentarization and tend to work in salaried professions, free trades, or day labor. Vestiges of nomadic life may remain, such as tents next to fixed permanent dwellings.
- *Spontaneous settlements in the vicinity of permanent settlements*: Bedouins living on the outskirts or close to permanent settlements where limited services, such as health and education, are offered. Bedouins in this group may retain aspects of tribal economy behavior, such as raising herds and nomadic agriculture, in addition to cash jobs and may live in clans or subclans.
- *Peripheral settlement*: Bedouins geographically distant from any permanent settlement. This life style is semi-nomadic with living structures including tents or shacks. This group has no access to modern services at its settlement locality and the tribal behavior is the hallmark at this stage.

Today, almost 60% of the Negev Bedouins live in planned villages and cities. The rest reside in the shantytown-like spontaneous settlements, with less than 10% continuing the traditional semi-nomadic life style.

The Negev Bedouins have the lowest overall socioeconomic status of any social or ethnic group in Israel[2]. They have half the per capita income, twice as many children, and half the living space compared to the average Israeli. Rates of unemployment and welfare support are among the highest in Israel. Bedouin students complete secondary school matriculation exams at less than half the rate of their Jewish neighbors in the Negev or Israeli Arabs in general[2]. The low status of women and children, a common characteristic of Middle Eastern tribal traditions, still has a major impact on the lives of these groups.

Rahat is the largest Bedouin settlement in the northern Negev. At the end of 2002, there were 19,900 children in Rahat. The percentage of children (61.3%) out of the whole population is more than double that of other cities in Israel.

## CHILDHOOD INJURY

Unintentional injury is the number one cause of death in the 1- to 35-year life span[5]. Each year, many children lose their lives due to causes that were preventable with reasonable resources[6]. In Israel, as well as in other countries, accidental injuries are considered a major public health concern that affect morbidity, hospitalization, and mortality of children at all ages, both sexes, Jews and non-Jews. Furthermore, accidents are a main factor in disability and loss of potential life years. One of the important intervention strategies for injury prevention is environmental modification and changes; for example, removing hazards in the home and neighborhood to create a safer environment. Creating a safer environment is considered to be more efficient than explanatory intervention through health education alone[7].

The Bedouin children in the Negev are at a much higher risk for unintentional injuries. This can be explained by many factors, mainly due to an unsafe environment with no playgrounds, very low socioeconomic status, large families often with 10–14 children, and cultural differences. Despite the dismal picture, accidental injuries in Bedouin children can be prevented and are not inevitable.

## METHODS IN THIS STUDY

This intervention study took place between March 2003 and March 2004 in the Bedouin city of Rahat in the Negev region of Israel. The main goal was to identify hazards and dangerous obstacles in public places in Rahat, then remove or repair the obstacles found, in order to secure a safe public environment for children. “Obstacle” was defined as any barrier that could endanger the safety of a child.

The prevention program was based on a local coalition headed by the mayor. Coalition members represented the health sector, law enforcement, fire department, education, politicians, and welfare and social workers. High-school students were trained in childhood injury prevention and their tasks included the detection of safety obstacles in public places and their removal.

The ways of detecting the obstacles and the ways of managing or removing them changed according to reality and the responsibility of different authorities regarding the obstacle. The management of obstacles usually was done by turning to the authorities who were responsible for the area. When the obstacle was under the responsibility of the sanitation department of the municipality, an immediate report was given. When the obstacle was under the responsibility of different contractors of the city (pavement contractors, sewage contractors, etc.), the management became complicated and required more serious intervention. Furthermore, there were obstacles near living residences in a public area, but of private responsibility, so the report was given to the owner of the residence.

The assessment, intervention, and prevention program was performed by a local “Beterem” coordinator living in the community of Rahat. She started out as a volunteer guide on child safety at the local child development center and in an urban renewal project with the Municipal Department of Social Affairs. She was instructed by a steering and research committee of professionals for this intervention program.

The goal of the intervention program was the removal of at least 30% of environmental obstacles/hazards. The intervention program targets were evaluation of the intervention program execution and evaluation of the program achievements.

The evaluation method was performed according to the reports of the coordinator regarding the ways, processes, and problems encountered when handling the obstacles during the whole year of the program. The purpose of assessing the results was (1) to evaluate if the program succeeded in removing the obstacles and if it achieved its goals, and (2) to persuade policy makers to keep supporting the project. Evaluation of the results was based on the reports and documentation, and on filming the obstacle pre- and postmanagement. Any obstacle repaired or removed from the children’s living environment was defined as a case of success. Evaluation was done also by the “quality” method. Every obstacle was documented and checked on its own by content analysis. During the year of intervention, phone surveillance was conducted by the research committee with the coordinator in Rahat, after the detection of obstacles, to discuss the way of managing them and the difficulties encountered, if any, with the authority in charge.

All environmental hazards near living residences and public places in Rahat were documented. Safety hazards inside houses or in private territories were not included. The hazards documented were permanent environmental hazards endangering everybody. The hazard sampling was performed in three ways: touring through the neighborhoods and searches for hazards, arriving to a place accidentally and noticing existing hazards, and through lectures and talks with mothers at the local child development center.

## RESULTS

Table 1 presents the hazards found in Rahat according to description of the hazard, location, date of handling, and the way of management. Figs. 1–10 show the hazards before and after. According to the table, one can learn about the way of handling every one of the hazards. The results in Table 1 show that out of 10 hazards, 8 were removed.

## DISCUSSION

This 1-year study describes the method of locating hazards and removing or repairing them. The research findings showed the ways of detecting and managing these hazards, the process, and the difficulties in the handling. Our findings showed that the intervention program achieved the goal to remove at least a third of the hazards found. Of the hazards, 80% were successfully removed, while the management of the two remaining hazards began, but did not end, in the defined period for assessment.

The successful management required a number of factors: a personal involvement and sense of responsibility of a coordinator accompanied by a group of active volunteers, high community empowerment and involvement, and professional, strong national support. The project succeeded only because it had a personal value for the coordinator herself. The coordinator had dealt with health education in the area for 7 years and knew the residents well and saw things as a resident, a neighbor, and as a mother. She is known from the child development center and is familiar with the community's language, rules, and culture.

She used several ways to handle the issues at hand, like:

- *Referral to several authorities*. The coordinator kept on with the work, even when she was denied by some, in order to find the responsible authority. When she encountered an inappropriate factor, she applied to another until she detected the responsible authority. The ability to overcome "sealed ears" was needed in order not to leave the hazard unattended for a long time.
- *The power of warning and persuasion* by imposing liability for a disaster if the hazard would not be corrected. ("When the child becomes hurt, you will have to prove that it is not your fault.")
- *Sensitivity* towards the responsible authority by imposing liability and also emphasizing the sensitivity of the matter from the responsible side. ("If your nephew or child were hurt, how would you feel?")
- *High personal involvement,* as in one event when she gave the owners her own private hose. This action shows personal willingness, responsibility, and involvement.
- *Alertness to responsibility "attack",* where all the relevant factors required as evidence were presented at the right place and at the right time to the responsible authority; whether by writing letters, presenting documents or photographs, and persuasion through the child development center staff.
- *Short handling time* (1, 2 weeks) in order to bring the responsible authority to start taking care of the issue quickly. The responsible authority often turned out to be an intellectual who understood the danger imminent to the children with the coordinator only enlightening them.

**TABLE 1**
**Safety Hazards for Children in the Bedouin City of Rahat, 2003–2004**

| | Description of the Hazard | Location | Duration to Solution | Date of Management | Way of Management |
|---|---|---|---|---|---|
| 1 | Sewer draining into a barrel in a playground (Fig. 1) | Neighborhood | 1 month | March 2003 | An appeal to the owners, who removed the barrel and added a sewer hose to the central sewage. The coordinator gave them her own hose (Fig. 2). |
| 2 | An old refrigerator standing on the sidewalk (Fig. 3) | Neighborhood | 2 weeks | April 2003 | An appeal to the owners to throw the refrigerator away. The owners claimed the municipality should remove it. An appeal to the sanitation department brought a solution (Fig. 4). |
| 3 | A defunct wall (Fig. 5) | Neighborhood | 1.5 months | May 2003 | The coordinator turned to the owners, who knew her from the child development center. They claimed to lack money to fix the wall, but instead removed the wall and planted trees as a fence (Fig. 6). |
| 4 | A diesel oil container open in a neighborhood | Neighborhood | 2 months | June 2003 | The container belonged to a grocery owner. Cars used it often. The smell spread throughout the neighborhood with a high risk of explosion. Two children were hospitalized after drinking from it and then delivery of oil stopped, but the container still remained. |
| 5 | A barrel closing a hole in the middle of the street | Entrance to the Rahat market | 3 months | June 2004 | First reference was to the nearest store. Afterwards referred to the sanitation department, who claimed they could not manage it. Then a letter to the mayor with a picture. Removal succeeded only after a seminar with the CEO of Beterem, who spoke with the mayor. |
| 6 | A hole in the middle of an urban sidewalk (Fig. 7) | Neighborhood | 1 month | September 2003 | The coordinator appealed to the municipality to find out who is responsible for the sidewalks in that neighborhood. She contacted the private contractor, but the matter was only fixed after a hard talk and fear of publication of negligence (Fig. 8). |
| 7 | Iron sticks inserted in the ground in an open territory on the way to school (Fig. 9) | Neighborhood | 1 week | October 2003 | The iron sticks were used for election flags. The territory serves children on their way to school. Appeal was made to the one in charge of propaganda and the iron sticks were removed and fenced in (Fig. 10). |

**TABLE 1 (continued)**

| | | | | | |
|---|---|---|---|---|---|
| 8 | Stray dogs around the city | Neighborhood | 1 week | March 2004 | A report to the municipality veterinarian. A nurse from the child development center and a child on his way back from school were bitten. The child was vaccinated against rabies and the vet killed the dogs. |
| 9 | Exposure to dangerous materials | Neighborhood | — | Not yet ended | A garage owner kept dangerous materials and tools in his yard. His own children and others were walking around the hazard. The coordinator tried persuading him to lock them away. She felt she did not succeed, since he claimed it is his job and that is the way he works. The coordinator will appeal to him again. |
| 10 | A bridge without a fence or divider (in winter there is a stream that one can drown in); sometimes children even swim there in the winter | Industrial area | — | Not yet ended | An appeal was made to the municipality. It is a territory that is thought to be outside the city area, but children who live close by go through there. The municipality said it was not their business. The municipality contractor arrived, but said it was not his responsibility. The subject is still being handled by the coordinator, who is trying to find out who is responsible for managing the hazard. |

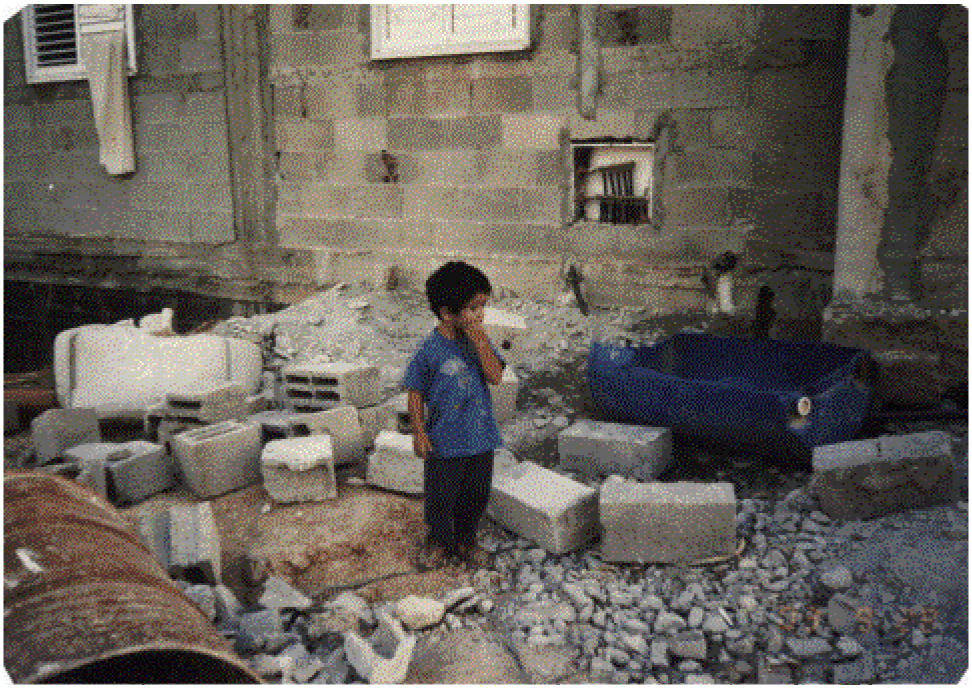

**FIGURE 1**. Sewer draining into a barrel in a playground.

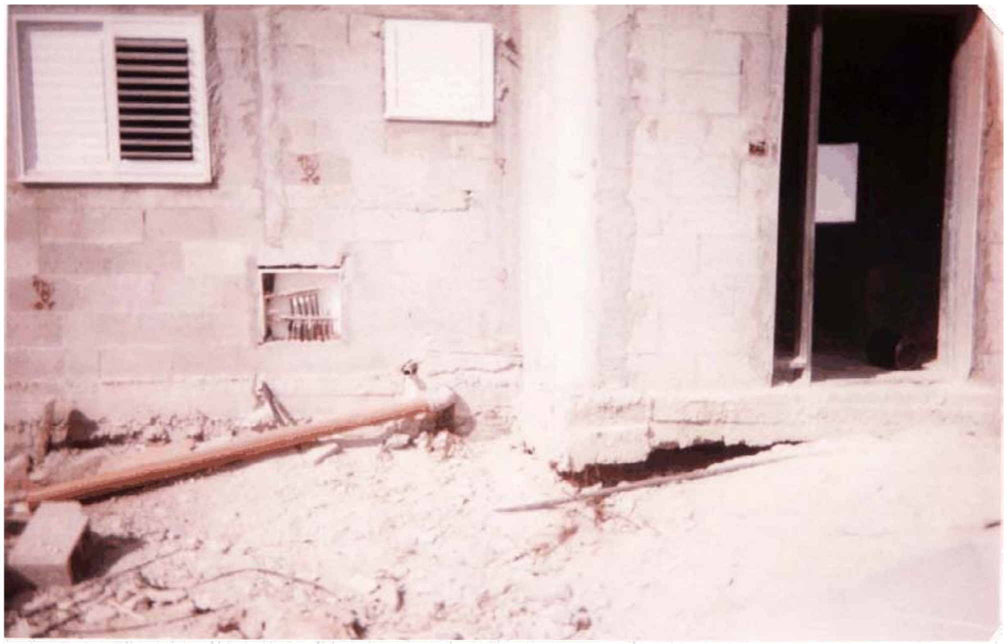

**FIGURE 2**. The sewage now connected to the main sewage system.

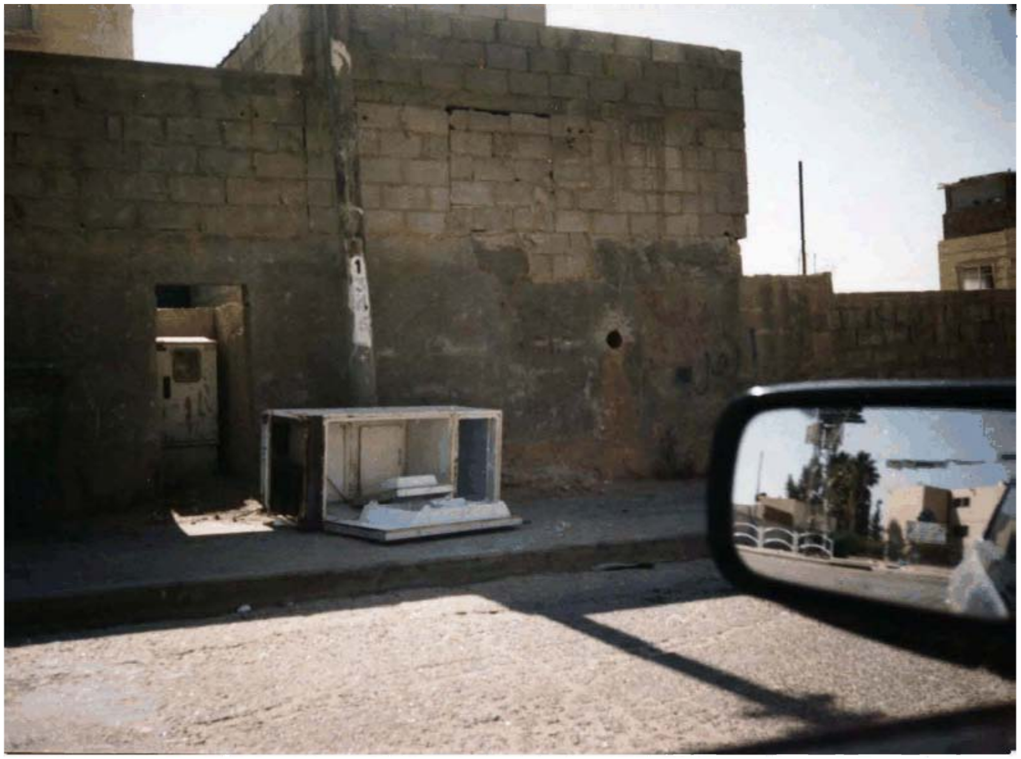

**FIGURE 3**. An open refrigerator on the sidewalk.

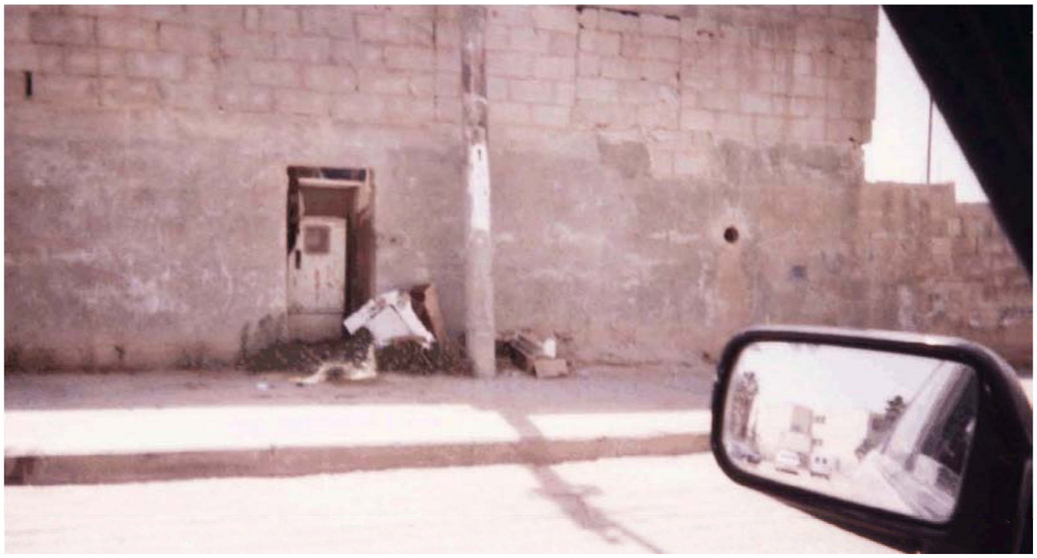

**FIGURE 4**. Removed after 2 weeks.

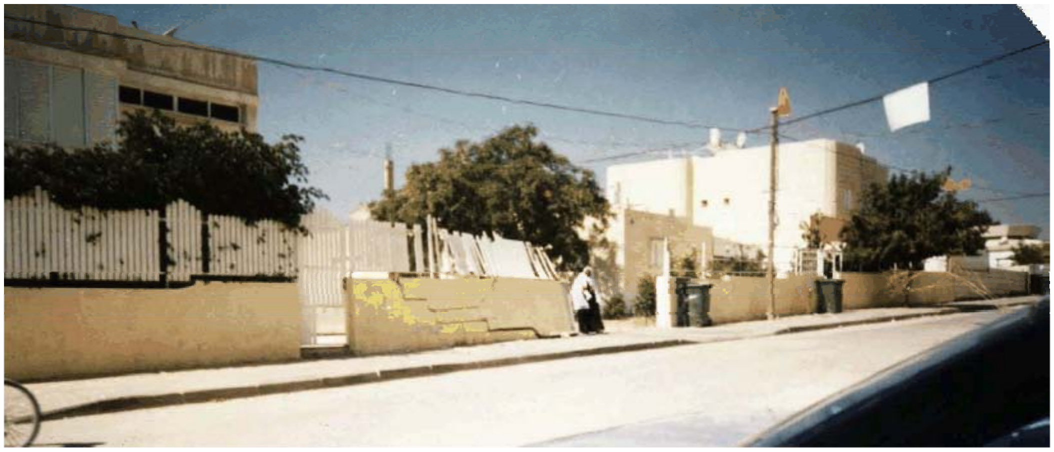

**FIGURE 5**. A wall towards the sidewalk in danger of falling.

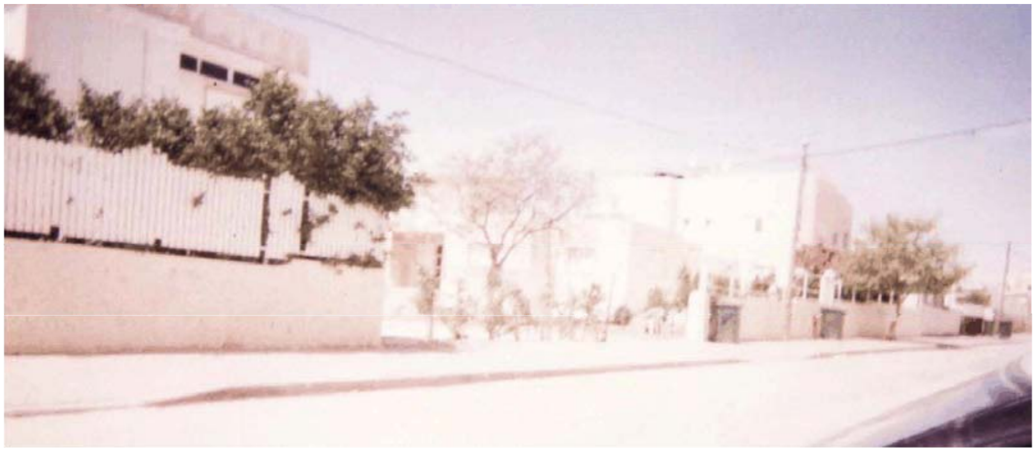

**FIGURE 6**. After 1.5 months, removed and trees planted.

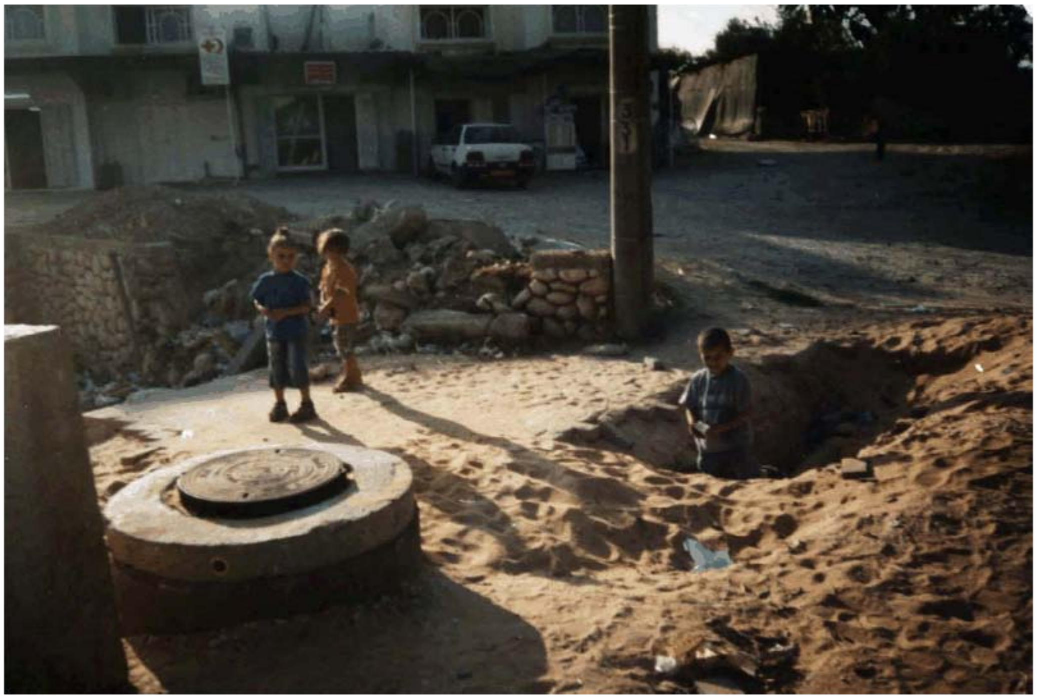

**FIGURE 7.** A hole in the middle of the sidewalk.

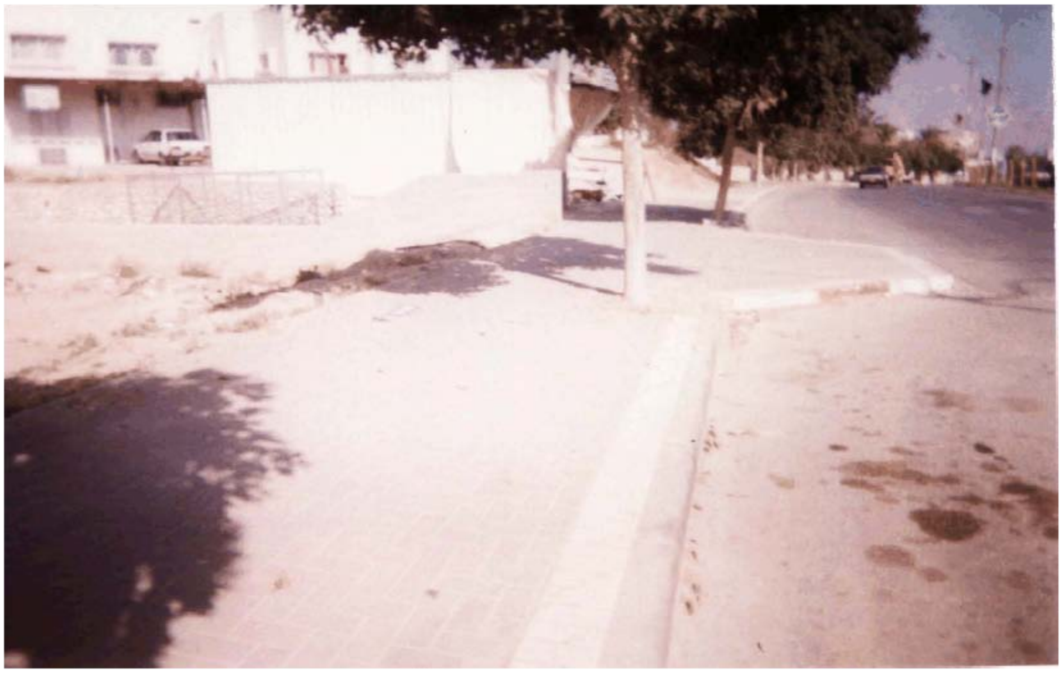

**FIGURE 8**. After 1 month, repaired.

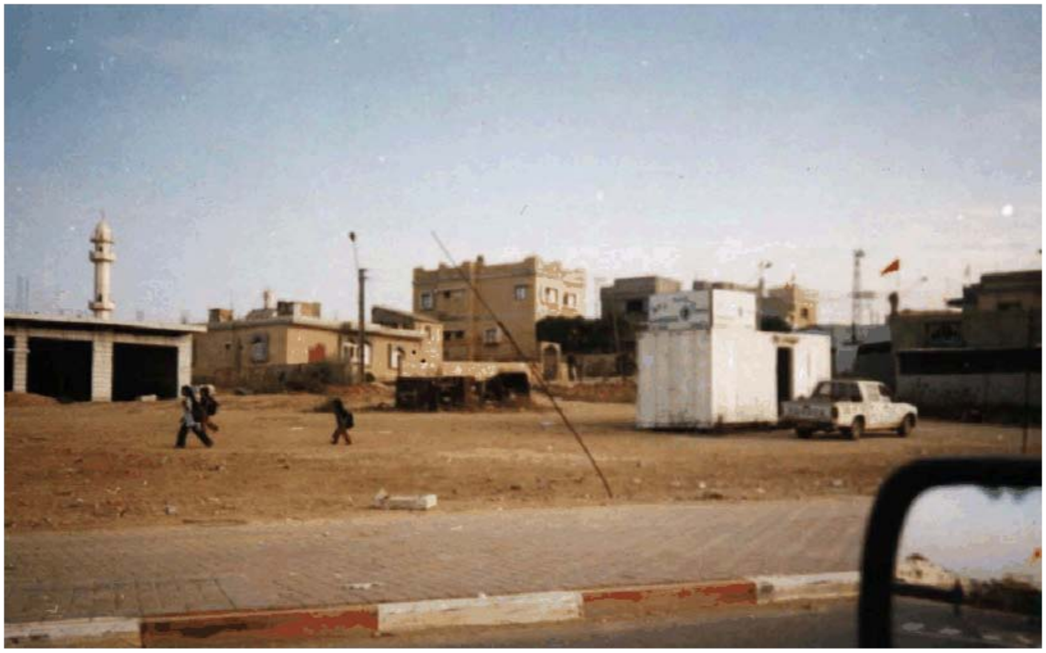

**FIGURE 9**. Iron sticks in the ground in an open area.

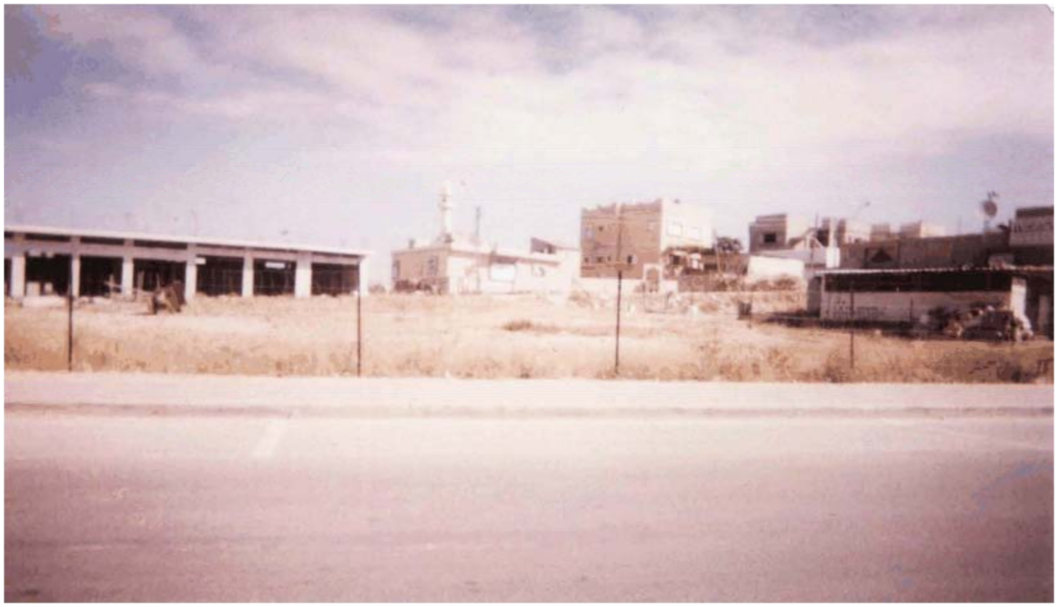

**FIGURE 10.** The sticks removed after 1 weeks and the area fenced in.

The seminar arranged in the community had a high level of attendence from the national Beterem organization, which helped to emphasize the work of the coordinator. The officials of the Beterem organization spoke with the mayor, sanitation executive, and others to obtain cooperation with the coordinator and stress the public interest of the issue at hand.

The intervention program achieved the goal to remove at least a third of the hazards found (in fact, 80%). Furthermore, agents were trained to handle the hazards themselves without the intervention of the coordinator, so in essence an educational element was added to the project. The safety changes were executed resulting in the sanitation and engineering departments now being able to handle these issues also and, today, pictures of hazards are used by the municipal authorities as documentation. There is now a close cooperation with the sanitation department in the municipality as opposed to the past. Hence, there are new agents for change acting on behalf of child safety and injury prevention. The unsuccessful management of the last two issues was due to the time frame of the project, but we believe that these hazards will be solved by the continuation of the work of the coordinator.

Difficulties reflected in the way of management were the feeling of inability to persuade people about the danger, not finding the responsible authority for the hazard, indifference of the responsible authority about their responsibility, misapprehension of the subject and the problem that children might get harmed, and problems on the cultural level.

There are a few limitations to this applied research, like a small sample of hazards, so it is difficult to draw statistical conclusions about the quantity of management and also the cultural aspect, which could make replication of this program elsewhere different.

## CONCLUSIONS

This study focused on public environmental hazards to child safety over a 1-year period by the involvement of a local safety coordinator in a Bedouin community in Israel. Most of the hazards (80%) detected and treated by the coordinator were solved, while the remaining are in the process of being solved. The exposure of the coordinator to the hazards was high for several reasons: she worked in the department of promoting safety in the community, her accumulated knowledge in safety brought her to look for hazards and watch them being managed as a natural part of her job, but also because she was part of the community herself.

It is recommended that an activity journal/diary and pictures ("a picture is worth a thousand words") be used to document the date of the hazard detection and the actions that were taken to solve the obstacle.

## ACKNOWLEDGEMENT

The research and the intervention were conducted by the support of Ashalim-Developing Services for Children at Risk and their Families (http://www.ashalim.org.il/).

## BIOSKETCHES

**Michal Hemmo-Lotem, MD**, is a pediatrician and a graduate of the School of Educational Leadership in Jerusalem. She has established Beterem-The National Center for Children's Health and Safety in Israel and is currently the director (CEO). Beterem dedicates its work to childhood injury prevention, safety, and research. She is the chairman of the National Committee for Childhood Injury Prevention and Safety and a member of the National Council for Child Health and Pediatrics, the National Council for Health Promotion, and is involved in the public debate on childhood injury and safety. She is a lecturer at the School of Public Health, Haifa University. E-mail: mhemmo@beterem.org

**Efrat Merrick, BA,** is a medical student at the Sackler School of Medicine, Tel Aviv University and a research assistant at the National Institute of Child Health and Human Development, Faculty of Health Sciences, Ben Gurion University of the Negev, Beer-Sheva, Israel. E-mail: efratmerrick@gmail.com

**Liri Endy-Findling, MPH,** educated at the School of Public Health at the Hebrew University is the Director of the Department of Research, Development and Policy at Beterem-the National Center for Children's Health and Safety, Israel. Beterem is an organization focused on injury prevention and safety promotion in childhood and Liri Endy-Findling is one of the leading figures in the organization. She has a rich background as a researcher from her experience at the National Research Institute for Social Sciences and Beterem. She leads public policy, programs development, research management, promotion of legislation and standards in the field of safety. E-mail: liri@beterem.org.il

**Aziza Abu Freh, BA**, is project coordinator for the Bedouin population in the south of Israel for Beterem, The National Center for Children's Health and Safety. She has extensive experience in education and community work and is a specialist in injury prevention. For the past years, she has been one of the leading figures for change in the awareness of injury prevention in the Bedouin community.

**Claudia Jinich-Aronowitz, RD, MPH,** is the Director of the Out-Reach Department in Beterem, The National Center for Children's Health and Safety in Israel. She is one of the founders of the organization and for the past 9 years has been dedicated to the promotion of safety, working with health and educational professionals at all levels, as well as with municipalities throughout the country. E-mail: claudiaja@beterem.org

**Liat Korn, MA** is a researcher at Beterem, the National Center for Child Safety and Health in Petach Tiqva, Israel and also a research associate in the Department of Sociology and Anthropology at Bar-Ilan University, Ramat Gan and organizer of the Israeli HBSC (Health Behavior in School-Aged Children) Study Group. Email: kornli@zahav.net.il

**Joav Merrick, MD, DMSc,** is professor of child health and human development affiliated with the Center for Multidisciplinary Research in Aging, Zusman Child Development Center, Division of Pediatrics and Community Health at the Ben Gurion University of the Negev, Beer-Sheva, Israel; the medical director of the Division for Mental Retardation, Ministry of Social Affairs, Jerusalem; and the founder and director of the National Institute of Child Health and Human Development, Ben Gurion University of the Negev, Beer-Sheva. Dr. Merrick has numerous publications in the field of child health and human development, rehabilitation, intellectual disability, disability, health, welfare, abuse, advocacy, quality of life, and prevention. He received the Peter Sabroe Child Award for outstanding work on behalf of Danish Children in 1985 and the International LEGO-Prize ("The Children's Nobel Prize") for an extraordinary contribution towards improvement in child welfare and well being in 1987. E-Mail: jmerrick@internet-zahav.net. Website: www.nichd-israel.com
